# ADCC responses and blocking of EGFR-mediated signaling and cell growth by combining the anti-EGFR antibodies imgatuzumab and cetuximab in NSCLC cells

**DOI:** 10.18632/oncotarget.17139

**Published:** 2017-04-17

**Authors:** Arjan Kol, Anton Terwisscha van Scheltinga, Martin Pool, Christian Gerdes, Elisabeth de Vries, Steven de Jong

**Affiliations:** ^1^ Department of Medical Oncology, University of Groningen, University Medical Center Groningen, Groningen, The Netherlands; ^2^ Department of Clinical Pharmacy and Pharmacology, University of Groningen, University Medical Center Groningen, Groningen, The Netherlands; ^3^ Roche Pharma Research & Early Development, Roche Innovation Center Zürich, Schlieren, Switzerland

**Keywords:** non-small cell lung cancer, EGFR, imgatuzumab, cetuximab, antibodies

## Abstract

Imgatuzumab is a novel glycoengineered anti-epidermal growth factor receptor (EGFR) monoclonal antibody optimized to induce both antibody-dependent cellular cytotoxicity (ADCC) and EGFR signal transduction inhibition. We investigated anti-EGFR monoclonal antibodies imgatuzumab and cetuximab–induced internalization and membranous turnover of EGFR, and whether this affected imgatuzumab–mediated ADCC responses and growth inhibition of non-small cell lung cancer (NSCLC) cells.

In a panel of wild-type EGFR expressing human NSCLC cell lines, membranous and total EGFR levels were downregulated more effectively by imgatuzumab when compared with cetuximab. Imgatuzumab plus cetuximab enhanced EGFR internalization and reduced membranous turnover of EGFR, resulting in an even stronger downregulation of EGFR. Immunofluorescent analysis showed that combined treatment increased clustering of receptor-antibody complexes and directed internalized EGFR to lysosomes. The antibody combination potently inhibited intracellular signaling and epidermal growth factor (EGF)-dependent cell proliferation. More importantly, robust EGFR downregulation after 72 hours with the antibody combination did not impair ADCC responses.

In conclusion, imgatuzumab plus cetuximab leads to a strong downregulation of EGFR and superior cell growth inhibition *in vitro* without affecting antibody-induced ADCC responses. These findings support further clinical exploration of the antibody combination in EGFR wild-type NSCLC.

## INTRODUCTION

The epidermal growth factor receptor (EGFR), a member of the human epidermal growth factor receptor (HER) family of receptor tyrosine kinases, is an important regulator of cell growth and differentiation. Upon ligand binding, EGFR homodimerizes or interacts with other HER members, i.e. HER2 and HER3, to form heterodimers. This results in activation of downstream signaling cascades such as the RAS-ERK pathway and PI3K/Akt pathway, thereby controlling many biological processes. These pathways are frequently dysregulated via overexpression, autocrine stimulation, crosstalk with other receptors and/or mutations, and play a pivotal role in multiple tumor types [1].

Targeting EGFR is an important treatment modality for many solid tumors including non-small cell lung cancer (NSCLC). Treatment strategies to target EGFR consist of tyrosine kinase inhibitors (TKIs) and monoclonal antibodies. At present, EGFR TKIs are standard of care for NSCLC patients with EGFR-mutated tumors as first- and second-line treatments [2]. Cetuximab (IgG1 subtype) is an anti-EGFR monoclonal antibody approved by the Food and Drug Administration USA for colorectal cancer and head and neck squamous cell cancer. Anti-EGFR monoclonal antibody panitumumab (IgG2 subtype), on the other hand, is approved for treatment of colorectal cancer. These monoclonal antibodies can exert their action via a variety of mechanisms, including blocking (hetero)dimerization and ligand binding, as well as inducing EGFR endocytosis, complement dependent cytotoxicity (CDC) and antibody-dependent cellular cytotoxicity (ADCC) [3–7]. ADCC is usually considered an important mechanism of action for immunotherapy with human IgG1 but not IgG2 antibodies. Recent evidence suggests that addition of cetuximab to chemotherapy in first line improves overall survival of patients with EGFR-expressing advanced NSCLC [8]. Interestingly, retrospective analyses revealed that mutational status and copy number of EGFR were not predictive for cetuximab benefit [9]. Therefore, treatment of EGFR wild-type NSCLC with anti-EGFR monoclonal antibodies might be of interest. Unfortunately, the impact of cetuximab on survival is small and the prognosis of advanced NSCLC remains poor. There is a continuous need for new treatments to improve survival. A stronger inhibition of EGFR or higher level of ADCC can potentially contribute to this improvement.

Anti-EGFR monoclonal antibody combinations can enhance endocytic downregulation of EGFR, and these combinations showed superior anticancer efficacy in several human tumor xenograft models, including triple negative breast cancer and EGFR-mutant NSCLC [10–12]. A higher level of ADCC can be achieved by glycoengineering the Fc region of a therapeutic antibody. Imgatuzumab (GA201) is a novel glycoengineered anti-EGFR monoclonal antibody of the IgG1 subclass, which is optimized to induce ADCC and inhibits EGFR signal transduction [13]. Imgatuzumab showed superior preclinical *in vivo* efficacy compared with cetuximab and non-glycoengineered imgatuzumab in both KRAS-mutant and KRAS wild-type models [13]. The clinical benefit of combining two monoclonal antibodies against EGFR is still unknown. Clinical benefit of combining antibodies has already been demonstrated for another HER family member, HER2, in breast cancer using the anti-HER2 antibodies trastuzumab and pertuzumab [14–16]. Trastuzumab binds to HER2 and suppresses its signaling capability. Pertuzumab complements the mechanism of action of trastuzumab by binding to another epitope of HER2, which inhibits the dimerization of HER2 with other HER receptors.

Imgatuzumab and cetuximab are directed against distinct, non-overlapping epitopes in EGFR extracellular domain III [13]. Thus, the combination of both antibodies is a potential strategy to target EGFR more effectively than existing clinical single antibody treatments. It is unknown whether treatment with imgatuzumab or the combination with cetuximab increases EGFR internalization and/or reduces membranous turnover of EGFR in cancer cells, potentially diminishing ADCC responses. The aim of the present study was therefore to investigate the effects of imgatuzumab and cetuximab on EGFR dynamics, intracellular signaling and survival in a panel of human EGFR wild-type NSCLC cell lines. Finally, we monitored whether changes in EGFR dynamics affect ADCC responses and tumor cell growth inhibition.

## RESULTS

### Imgatuzumab combined with cetuximab strongly downregulates EGFR expression in NSCLC cells

**Figure 1:**
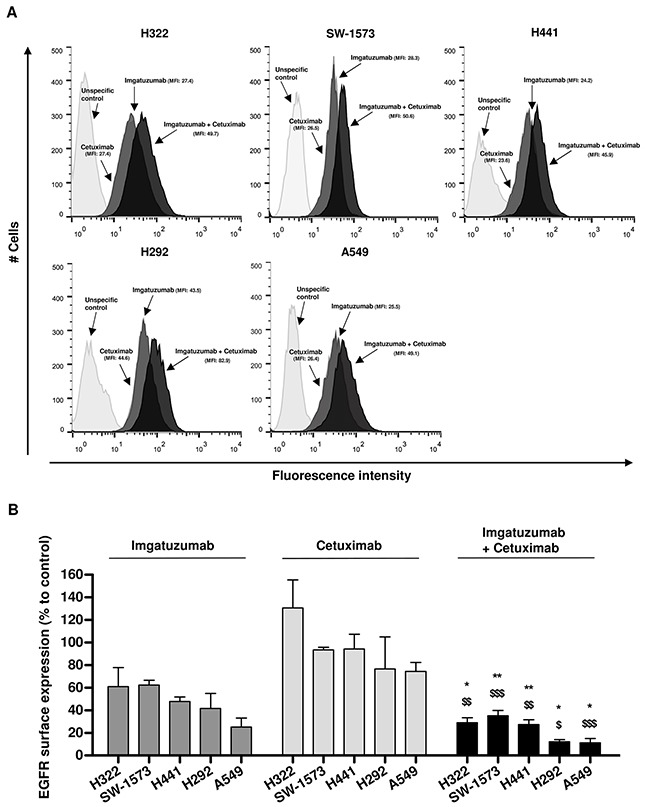
Effect of anti-EGFR monoclonal antibody treatment on EGFR surface expression levels **(A)** Flow cytometric analysis of imgatuzumab and cetuximab binding alone or in combination in H322, SW-1573, H441, H292 and A549 cells. **(B)** H322, SW-1573, H292, H441 and A549 cells were treated with the anti-EGFR monoclonal antibodies (20 μg/mL total) for 72 hours. Surface expression levels were determined using flow cytometry. The surface expression in untreated control cells was set at 100% both for the single antibodies and the combination. (*P < 0.05, **P < 0.01 combination vs imgatuzumab; ^$^*P* < 0.05, ^$$^*P* < 0.01, ^$$$^*P* < 0.001 combination vs cetuximab; unpaired t-test). Data points are mean + SD (n = 3).

All NSCLC cell lines expressed EGFR, with the highest cell surface levels found in H292 cells (Figure 1A). Addition of cetuximab to imgatuzumab resulted in a nearly two-fold increase in mean fluorescence intensity of membranous EGFR (Figure 1A), which is in line with previous findings that imgatuzumab and cetuximab are binding to non-overlapping epitopes in EGFR extracellular domain III [13]. Next, we measured EGFR levels following incubation of cells with imgatuzumab and cetuximab alone or combined for 72 hours. In the presence of imgatuzumab, membranous EGFR levels were diminished by 38% in SW-1573 and up to 75% for A549, whereas cetuximab had less effect (up to 26% for A549) (Figure 1B). Treating cells with the combination of monoclonal antibodies resulted in a stronger downregulation of membranous EGFR levels ranging from 65% in SW-1573, up to 89% for A549. Similar results were observed with 24 hours incubation or twice the amount of each monoclonal antibody (Supplementary Figure 1A), which suggests an equilibrium in membranous turnover of EGFR. A non-glycoengineered GA201 (GA201_wt_) was used to investigate the effect of antibody glycoengeneering on EGFR surface expression. GA201_wt_ and imgatuzumab had similar effects on membranous EGFR in SW-1573 and H292 cells, excluding the involvement of glycoengeneering (Supplementary Figure 1B).

**Figure 2:**
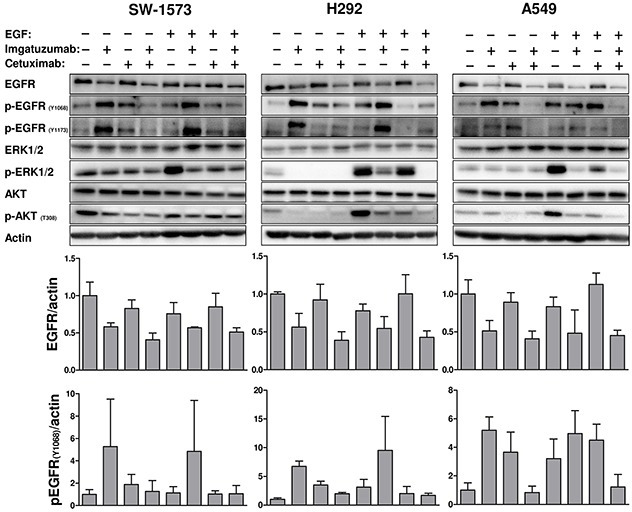
Western blot analysis of the effect of 24 hours anti-EGFR monoclonal antibody treatment on EGFR total protein levels and downstream signaling under normal growth conditions or 15 min EGF stimulation (10 ng/mL) The immunoreactive spots were quantified by densitometric analysis and normalized using actin (see also Supplementary Figure 2). Values are expressed as fold increase versus control + SD. All experiments were performed in triplicate.

Western blot analyses demonstrated that treatment of SW-1573, H292 and A549 cells with imgatuzumab alone or combined with cetuximab led to a decrease in total cellular EGFR protein levels as well (Figure 2). Both single agents and the combination efficiently inhibited EGF-induced phosphorylation of downstream signaling molecules such as Akt and ERK1/2 (Figure 2 and Supplementary Figure 2). In H292, only the combination was able to completely inhibit EGF-induced Akt and ERK1/2 phosphorylation. Interestingly, treatment with imgatuzumab or cetuximab increased EGFR phosphorylation at Tyr1068 and Tyr1173, but did not lead to increased phosphorylation of Akt or ERK1/2. Despite a lower level of phosphorylated EGFR at both phosphorylation sites, EGF was able to activate EGFR downstream signaling in contrast to imgatuzumab or cetuximab. Addition of cetuximab to imgatuzumab counteracted the increase in EGFR phosphorylation. GA201_wt_ and imgatuzumab had similar effects on EGFR protein levels and phosphorylation in SW-1573 and H292 cells (Supplementary Figure 3A).

Taken together these results indicate a strong reduction in EGFR cell surface expression and total cellular EGFR expression by continuously exposing cells to imgatuzumab, which was not observed with cetuximab. Combining imgatuzumab with cetuximab augments the reduction in EGFR expression.

### Imgatuzumab combined with cetuximab enhances EGFR internalization and reduces EGFR turnover at the plasma membrane

**Figure 3:**
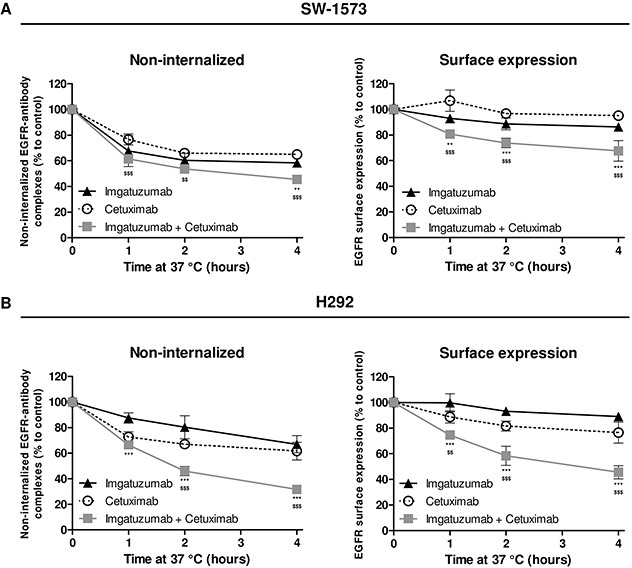
Influence of monoclonal antibody binding on EGFR internalization and surface expression SW-1573 **(A)** and H292 cells **(B)** were surface labeled with the anti-EGFR monoclonal antibodies on ice and incubated at 37°C for the indicated times, and then analyzed for the non-internalized EGFR-antibody complexes (left) and cell surface expression (right). The lower the amount of non-internalized EGFR-antibody complexes, the higher the amount of internalized antibody-EGFR complexes. The surface expression at t=0 was set at 100%. The non-internalized and surface expression were determined as described in *Materials and Methods (n=3)*. Data points are mean ± SD. (**P < 0.01, ***P < 0.001 combination vs imgatuzumab; ^$$^*P* < 0.01, ^$$$^*P* < 0.001 combination vs cetuximab; two-way ANOVA followed by Bonferroni post-test).

Flow cytometry was used to determine whether the downregulation of EGFR is due to enhanced internalization of this receptor. SW-1573 and H292 cells were pre-loaded with imgatuzumab and cetuximab on ice and subsequently incubated at 37°C for the indicated time points. Experiments with radiolabeled monoclonal antibodies showed a minimal release of monoclonal antibodies from cells during 4 hours (results not shown), indicating that antibody internalization is a measurement of EGFR internalization. Imgatuzumab and cetuximab were internalized at similar rates in SW-1573 (Figure 3A) and H292 cells (Figure 3B), as demonstrated by a decrease in non-internalized EGFR-antibody complexes. GA201_wt_ and imgatuzumab had similar effects on EGFR internalization and surface levels in SW-1573 and H292 cells (Supplementary Figure 3B). Despite the rapid internalization of these complexes, overall EGFR cell surface levels marginally changed by single antibody treatment, indicating that membranous turnover of EGFR was not rate limiting within this timeframe. Imgatuzumab in combination with cetuximab, however, accelerated EGFR internalization in SW-1573 and H292 cells, which resulted in a strong reduction of overall EGFR cell surface levels. Pretreatment with the protein synthesis inhibitor cycloheximide reduced basal EGFR surface levels. However, the percentage internalized antibody–EGFR complexes and the percentage EGFR surface expression on SW-1573 and H292 cells after 2 hours incubation with antibodies at 37°C were proportionally similar to the results in antibody-treated cells without cycloheximide pretreatment (results not shown). These findings indicate that, within this 2 hours timeframe, the absolute level of membranous EGFR was affected by cycloheximide treatment, whereas the kinetics of antibody-induced internalization and (re)appearance of EGFR on the cell surface was predominantly determined by the antibody used.

### EGFR tyrosine kinase inhibition by erlotinib does not affect EGFR internalization and degradation by imgatuzumab combined with cetuximab

**Figure 4:**
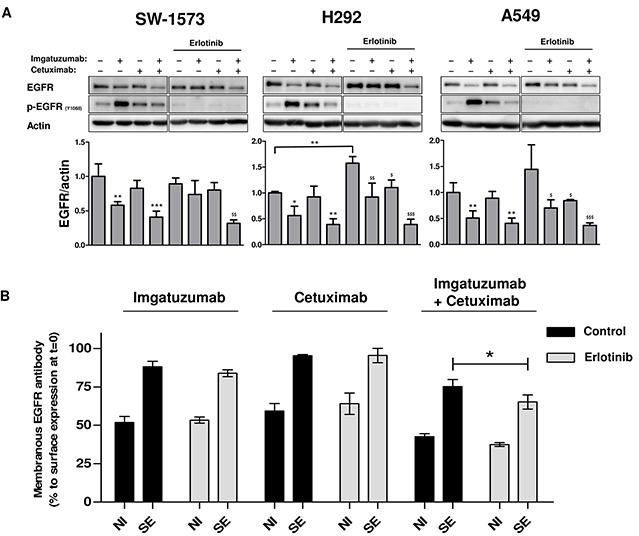
Effects of TKI treatment on monoclonal antibody-induced internalization and degradation **(A)** Western blot analysis of the effect of 24 hours anti-EGFR monoclonal antibody treatment (20 μg/mL total) with or without erlotinib (10 μM) on EGFR total protein levels in SW-1573, H292 and A549. Data points are mean + SD. (*P < 0.05, **P < 0.01, ***P < 0.001 compared to untreated control; ^$^*P* < 0.05, ^$$^*P* < 0.01, ^$$$^*P* < 0.001 compared to erlotinib treated control). **(B)** SW-1573 cells were pretreated for 24 hours with the EGFR TKI inhibitor erlotinib (10 μM). Cells were subsequently surface labeled with the anti-EGFR monoclonal antibodies (20 μg/mL total) on ice and incubated at 37°C for 4 hours, and then analyzed for the non-internalized EGFR-antibody complexes (NI) and cell surface expression (SE). The lower the amount of non-internalized EGFR-antibody complexes, the higher the amount of internalized antibody-EGFR complexes. Surface expression of the control and erlotinib treated cells were both set at 100%. 24 hours erlotinib treatment resulted in a slight upregulation (10 – 15 % increase). Data points are mean ± SD (n = 3). (*P < 0.05).

We sought to determine whether imgatuzumab-induced EGFR phosphorylation is instrumental in the strong reduction in EGFR protein levels. SW-1573, H292 and A549 cells were treated with erlotinib to inhibit EGFR tyrosine kinase activity. Co-treatment of cells with erlotinib and the monoclonal antibodies individually for 24 hours showed that erlotinib indeed reduced p-EGFR levels (Figure 4A). Interestingly, erlotinib inhibited imgatuzumab-induced EGFR protein degradation, but had no effect on EGFR degradation by the antibody combination. It should be noted that erlotinib treatment alone increased EGFR expression in H292 and A549 cells, which makes these results more difficult to interpret. To investigate the influence of EGFR kinase activity on monoclonal antibody-induced internalization in a short-term experiment, SW-1573 cells were pretreated for 24 hours with erlotinib. Erlotinib did not reduce internalization of antibody-EGFR complexes (Figure 4B). In fact, total EGFR surface expression was significantly reduced when the antibody combination was added to erlotinib-pretreated cells. These results demonstrate that p-EGFR inhibition is not antagonizing EGFR internalization and degradation induced by the antibody combination.

### Imgatuzumab combined with cetuximab increases clustering of EGFR-antibody complexes and targets EGFR to the lysosomal degradation pathway

**Figure 5:**
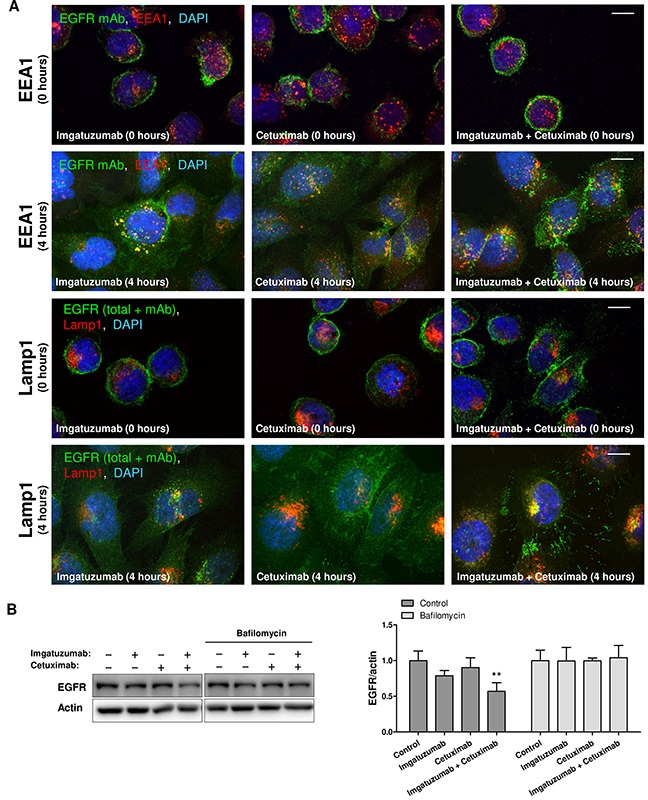
Cellular localization and endocytic trafficking of anti-EGFR monoclonal antibodies in SW-1573 **(A)** Cells were surface labeled with the anti-EGFR monoclonal antibodies (mAbs) on ice. After staining with the primary antibody cells were washed with ice-cold FACS buffer and incubated for 4 hours at 37°C. After fixation, permeabilization, and incubation with anti-EEA1 and anti-Lamp1 antibodies, cells were incubated with fluorescent secondary antibodies (anti-human Alexa 488 against imgatuzumab and cetuximab (green color), Alexa 647-labeled antibodies for endosomal markers (red color)). For the Lamp1 staining additional anti-EGFR monoclonal antibodies were added to visualize total EGFR. Colocalization appears as yellow (merged images). Images were acquired using a confocal microscope. White scale bars represent 10 μm. **(B)** Western blot analysis of the effect of lysosomal inhibition on monoclonal antibody-induced EGFR degradation. SW-1573 cells were pretreated for 2 hours with the lysosomal inhibitor bafilomycin A1 (100 nM). Cells were subsequently treated with the anti-EGFR monoclonal antibodies (20 μg/mL total) for 4 hours. Data points are mean + SD. (**P < 0.01 compared to control).

The intracellular localization of EGFR after antibody treatment was investigated with immunofluorescent microscopy, focusing on the endocytic and lysosomal degradation pathways. Staining of SW-1573 cells with imgatuzumab and/or cetuximab as primary antibodies clearly showed membranous EGFR fluorescence (Figure 5A). SW-1573 and H292 cells were subsequently incubated with the anti-EGFR monoclonal antibodies on ice and then chased for 4 hours at 37°C. Imgatuzumab and cetuximab localized to internal vesicles (Figure 5A and Supplementary Figure 4B). To exclude the possibility that the incubation with secondary antibodies triggers cluster formation due to crosslinking of monoclonal antibodies, SW-1573 cells were incubated with imgatuzumab directly labeled with Dylight 633. Cluster formation was still observed when imgatuzumab Dylight 633 was combined with cetuximab (Supplementary Figure 4A). We then stained SW-1573 and H292 cells for EEA1, a marker for early endosomes. Imgatuzumab and cetuximab mostly co-localized with EEA1-positive vesicles, indicating internalized monoclonal antibodies (Figure 5A and Supplementary Figure 4B). Following incubation of cells for 4 hours at 37°C, the antibody combination localized to large clusters at the surface and/or intracellular vesicles (Figure 5A and Supplementary Figure 4B). In cells treated with imgatuzumab combined with cetuximab, antibody-positive vesicles were only partially co-localizing with EEA1-positive vesicles, suggesting that the EGFR antibody combination complexes are localized in a different compartment. Therefore, cells were stained for LAMP1 to identify the lysosomal compartment. The monoclonal antibodies did not co-localize with lysosomes (data not shown), which may be due to degradation of receptor antibody-complexes in lysosomes. Adding additional anti-EGFR monoclonal antibodies after fixation showed that EGFR predominantly co-localized with lysosomes in cells treated with both imgatuzumab and cetuximab (Figure 5A and Supplementary Figure 4B). Blocking lysosomal proteolysis by bafilomycin A1 largely prevented monoclonal antibody combination-induced EGFR degradation in SW-1573 (Figure 5B) and A549 cells (Supplementary Figure 4C). However, EGFR degradation by monoclonal antibody combination treatment was only partially prevented in H292 cells (Supplementary Figure 4C). Taken together, these results show that the strong downregulation after treatment with imgatuzumab alone and combined with cetuximab is at least partially the result of directing EGFR to lysosomes. These observations are consistent with findings of Ferraro *et al*. showing that anti-EGFR monoclonal antibody mixtures accelerate lysosomal degradation of EGFR [12].

### Imgatuzumab combined with cetuximab effectively inhibits EGF-dependent cell proliferation

**Figure 6:**
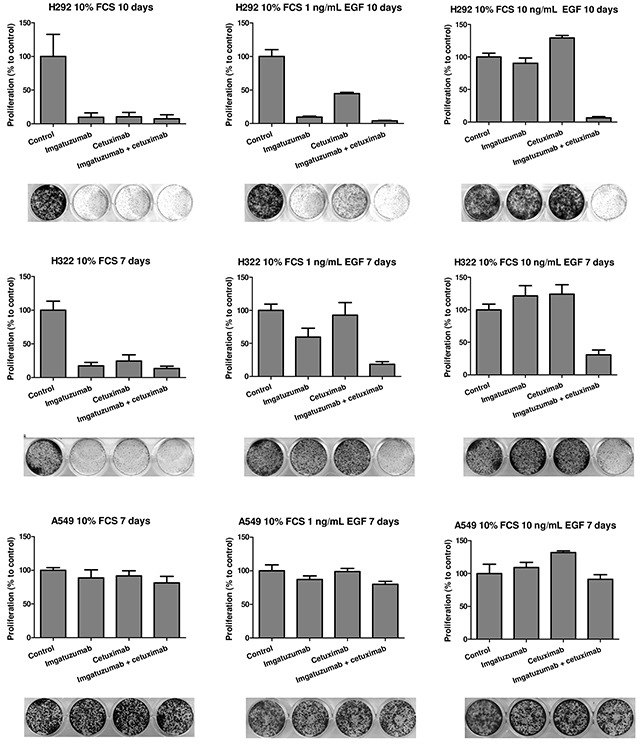
*In vitro* growth-inhibitory effect of anti-EGFR monoclonal antibody treatment Cells were seeded in 12-wells plates and allowed to adhere overnight. Next, H292, H322 and A549 cells were treated with the anti-EGFR monoclonal antibodies (20 μg/mL total) for the indicated days under normal growth conditions without additional EGF (medium with 10% FCS) or in medium containing 10% FCS with 1 or 10 ng/mL EGF. Data points are mean + SD. All experiments were performed in triplicate.

Next, the antiproliferative effect of imgatuzumab and cetuximab on SW-1573, H292, H322, H358 and A549 cells was investigated. Under normal culture conditions (media with 10% FCS), both single agents and the combination strongly inhibited H292 and H322 cell proliferation (Figure 6). The antiproliferative effects in H292 correlated with strong inhibition of downstream signaling after monoclonal antibody treatment (Figure 2). To investigate the growth inhibitory potential of the monoclonal antibodies in the presence of additional EGF, H292, H322 and A549 cells were incubated with imgatuzumab, cetuximab or the combination under normal culture conditions supplemented with physiological concentrations of EGF. Imgatuzumab was more effective than cetuximab in inhibiting proliferation in media supplemented with 1 ng/mL EGF. Addition of 10 ng/mL EGF to media induced complete resistance to the single antibody treatments, whereas the antibody combination was still able to almost completely inhibit proliferation. As expected, no clear effects of imgatuzumab and cetuximab on proliferation were observed in KRAS-mutant A549 (Figure 6) and SW-1573 cells (results not shown). Interestingly, under normal growth conditions and supplemented with 1 ng/mL EGF, imgatuzumab alone and combined with cetuximab partially inhibited proliferation of KRAS-mutant H358 cells (Supplementary Figure 5). Under conditions of high EGF, only the combination inhibited proliferation.

Both single antibody treatments and the combination completely inhibited EGF-dependent migration of H292 and A549 cells as measured with the wound-healing assay (Supplementary Figure 6).

### Imgatuzumab combined with cetuximab induces strong ADCC but no CDC responses against NSCLC cells

**Figure 7:**
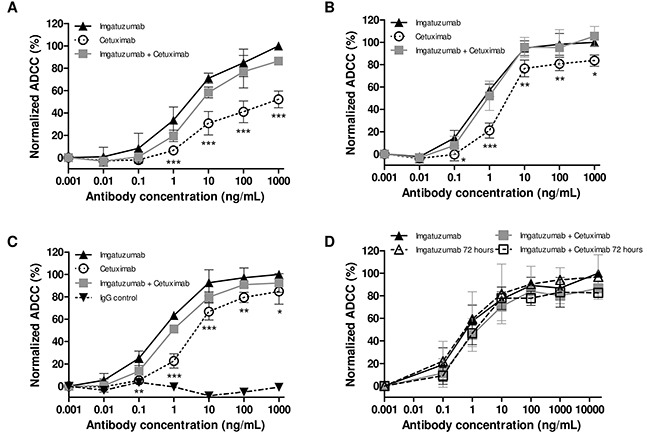
*In vitro* ADCC analysis of imgatuzumab, cetuximab or the combination PBMCs as effector cells were added to the untreated H441 **(A)**, H292 **(B)** or A549 **(C)** target cells at a ratio of 25:1. ADCC responses were normalized to the ADCC response of the highest imgatuzumab concentration due to variations in responses between PBMCs of different donors. Absolute ADCC responses ranged between 12 and 53%. (*P < 0.05, **P < 0.01, ***P < 0.001 cetuximab vs imgatuzumab). **(D)** A549 cells were pretreated with imgatuzumab alone (20 μg/mL) or combined with cetuximab (20 μg/mL total) in culture medium for 72 hours. Next, cells with (open triangle and open square) or without (solid triangle and solid square) antibody pretreatment for 72 hours were washed and the ADCC assay was performed using PBMCs as effector cells and A549 cells as target cells at a ratio of 25:1. Data points are mean ± SD (n = 3).

Imgatuzumab combined with cetuximab is the most potent in downregulating EGFR surface expression. However, effective EGFR downregulation might impair imgatuzumab-dependent ADCC responses. Alternatively, imgatuzumab and cetuximab are noncompetitively binding to EGFR (Figure 1A), which may even result in stronger ADCC responses. We performed an *in vitro* ADCC assay, in which human peripheral blood mononuclear cells (PBMCs) were used as effector cells and H441, H292 or A549 cells as target cells. When applied as single agent, imgatuzumab caused superior ADCC against NSCLC cells compared with cetuximab (Figure 7A-7C). No increase in ADCC responses was observed when the two monoclonal antibodies were combined. Next, the effect of EGFR downregulation on the ADCC response after long-term treatment with imgatuzumab was investigated in A549 cells. These cells were chosen based on the strong downregulation of membranous EGFR (Figure 1B) and their resistance to anti-EGFR monoclonal antibody treatment under normal growth conditions to avoid anti-proliferative effects (Figure 6). Pretreatment of cells with imgatuzumab for 72 hours did not alter ADCC responses despite the strong reduction in membranous EGFR (Figure 7D). Similar results were observed when cells were treated with imgatuzumab combined with cetuximab for 72 hours (Figure 7D).

Next, we investigated whether imgatuzumab and cetuximab induce CDC in our NSCLC cell line panel. For the *in vitro* CDC assay freshly drawn human serum was used. CDC was not induced in H292 and A549 with imgatuzumab or cetuximab, whereas a CDC response was observed in EGFR-amplified squamous-cell carcinoma A431 cells as positive control (Supplementary Figure 7). Addition of imgatuzumab to cetuximab significantly enhanced a CDC response in A431 cells only.

## DISCUSSION

In the present study we show that glycoengineered imgatuzumab downregulated membranous and cellular EGFR protein levels, whereas cetuximab had almost no effect in a panel of EGFR wild-type expressing NSCLC cell lines. Dual EGFR targeting with imgatuzumab in combination with cetuximab led to a stronger downregulation of membranous and cellular EGFR protein levels by lysosomal targeting of the receptor, while ADCC effects were maintained. In addition, blocking EGFR with imgatuzumab combined with cetuximab most effectively inhibited cell proliferation. Our results suggest a novel strategy combining imgatuzumab with cetuximab for lung tumors expressing wild-type EGFR.

The ability of imgatuzumab to internalize and downregulate EGFR is not related to antibody glycoengeneering but might be partially attributed to the increased auto-phosphorylation levels of EGFR. Several anti-EGFR monoclonal antibodies applied as single agents act as partial agonists due to their ability to force EGFR homodimerization and subsequent auto-phosphorylation, leading to EGFR downregulation [12,17,18]. However, Gerdes *et al*. did not observe increased auto-phosphorylation levels after imgatuzumab treatment in A431 cells [13]. Furthermore, Cunningham *et al*., using the parental rat anti-EGFR antibody IRC62 and DiFi cells, showed that the antibody reduces EGFR phosphorylation at multiple sites [19]. These discrepancies might be due to differences in cell lines and experimental set-up. For example, both A431 and DiFi cells overexpress EGFR as a result of extensive *EGFR* gene amplification. Furthermore, A431 cells were cultured in spheroids instead of a 2D monolayer, which might have influenced dimerization and signaling. Even though imgatuzumab and cetuximab induced EGFR Tyr1068 and Tyr1173 phosphorylation in our experiments, it did not lead to increased phosphorylation of ERK1/2 and Akt. This is in line with previous findings that anti-EGFR antibodies, including cetuximab, can induce EGFR phosphorylation but fail to trigger downstream signaling in NSCLC and head and neck squamous cell carcinoma cell lines [17,18]. The absence of downstream signaling is also demonstrated by the fact that imgatuzumab was still capable of inhibiting cell proliferation with a similar potency as cetuximab. The inability of imgatuzumab and cetuximab to activate downstream signaling might be due to reduced antibody-EGFR complex internalization compared with ligand-EGFR complexes [17]. Probably, this interferes with signaling induced by internalized ligand-EGFR complexes, which can interact in the endosomal compartment with various downstream signaling proteins that are important for continuous activation of the major signaling pathways mediated by ERK [20, 21]. In addition, phosphorylation of EGFR on Tyr1173 might have a suppressive effect on ERK signaling [22]. However, we cannot exclude the possibility that increased auto-phosphorylation after imgatuzumab or cetuximab treatment activates other downstream pathways of EGFR with unknown effects on NSCLC cells [23]. Interestingly, the combination of imgatuzumab and cetuximab counteracted the increase in EGFR auto-phosphorylation levels observed after single antibody treatment and, therefore, might be the preferred strategy to inhibit EGFR signaling.

Our experiments with EGFR TKI erlotinib demonstrate that imgatuzumab-induced EGFR protein degradation, but not EGFR internalization is related to EGFR kinase activity. Surprisingly, erlotinib did not affect EGFR degradation when imgatuzumab was combined with cetuximab. This discrepancy could be the result of different endocytic pathways that were activated, which may depend on the size of receptor-antibody complexes, or receptor ubiquitination [24]. For example, formation of large EGFR–antibody complexes at the cell surface was found to internalize EGFR by macropinocytosis, resulting in efficient EGFR degradation [25]. In addition, it has been reported that increased cross-linking induced by monoclonal antibody combinations inhibits endosomal recycling to a much larger extent than single antibodies and accelerates lysosomal degradation of EGFR [10, 12]. Our results actually show that imgatuzumab combined with cetuximab increases clustering of receptor-antibody complexes and enhances (lysosomal) EGFR degradation, thereby reducing membranous turnover of EGFR.

ADCC has been described as a major mechanism of action for immunotherapy with human IgG1 molecules. The glycosylation status of an antibody, preferentially IgG1, is important for its ability to bind to Fcγ-receptors expressed on human immune effector cells, thereby enhancing the ADCC response [26, 27]. Since imgatuzumab does not interfere with binding of cetuximab on EGFR, the total amount of monoclonal antibodies on the cell surface is doubled when they are coincubated. Therefore, it was expected that in the ADCC assay, where human PBMCs, antibodies and low to moderate EGFR expressing tumor cells were coincubated, the antibody combination would lead to enhanced ADCC [13]. However, ADCC enhancement was not observed. In a second ADCC assay, tumor cells were preincubated with monoclonal antibodies. EGFR downregulation by long-term treatment with imgatuzumab with or without cetuximab, resulted in less antibody bound to the cell surface. Here, we observed that ADCC responses were not reduced. These results indicate that the threshold to induce the maximum amount of ADCC in this experimental setting is still maintained. The possibility that the antibody combination has different effects on monocyte and/or polymorphonuclear cell-mediated ADCC than single antibody treatment cannot be excluded based on these results. Our findings are supported by earlier *in vitro* work showing that the noncompetitive anti-HER2 monoclonal antibody combination pertuzumab plus trastuzumab reduced the amount of membranous HER2 on breast cancer cells, but maintained ADCC activity [14, 15]. Derer *et al*. also showed, using a stably transfected hamster kidney cell line overexpressing different EGFR cell surface levels and a panel of tumor cell lines with different EGFR cell surface levels, that maximal NK cell-mediated ADCC was already achieved at low membranous EGFR levels [28]. EGFR antibody combinations can enhance CDC activities in EGFR-amplified cell lines [7, 29]. However, we did not observe imgatuzumab-induced CDC in our NSCLC cell line panel expressing low to moderate membranous EGFR levels. Imgatuzumab clearly induced CDC in high EGFR expressing A431 cells, suggesting that membranous EGFR levels in our NSCLC cell line panel were too low for anti-EGFR antibody-induced CDC.

Even though certain EGFR IgG1 monoclonal antibody combinations such as Sym004 and MM-151 showed superior anticancer efficacy in several human tumor xenograft models, their effects on ADCC were only investigated in EGFR amplified cell lines [11, 29]. Effects of lower EGFR expression levels or strong membranous EGFR downregulation on ADCC were not investigated. Our results demonstrate that even after strong EGFR downregulation and concomitant proliferation inhibition, ADCC still provides a mechanism by which imgatuzumab in combination with cetuximab can provide additional antitumor effect, especially in EGFR and KRAS wild-type NSCLC. A recent study showed that ADCC activity of imgatuzumab compared with cetuximab is unaffected by conventional cancer premedication drugs and chemotherapy agents in various xenograft models [30]. Although Roche stopped its imgatuzumab clinical programme after it failed to meet its primary endpoint in a phase II trial in colorectal cancer [31], it will be of interest to explore the combination of imgatuzumab and cetuximab in such treatment strategies.

In conclusion, imgatuzumab in combination with cetuximab leads to stronger downregulation of EGFR and enhances the antitumor effect *in vitro*. Despite lowering EGFR surface levels, monoclonal antibody induced ADCC response is maintained. These findings support further clinical exploration of the antibody combination in EGFR wild-type NSCLC.

## MATERIALS AND METHODS

### Cells, antibodies and drugs

The human NSCLC cell lines SW-1573, H292, A549, H358 and H441, and the squamous-cell carcinoma A431 were obtained from the American Type Culture Collection (ATCC). H322 was obtained from Sigma-Aldrich. All cell lines are EGFR wild-type and H441, H358, SW-1573 and A549 are KRAS-mutant. Cells were quarantined until screening for microbial contamination and mycoplasma was performed and proven to be negative. Cells were tested and authenticated in April 2015 by Baseclear using STR profiling. NSCLC cell lines were cultured in RPMI media in the presence of 10% fetal calf serum (FCS) with (SW-1573, H322 and H441) or without 2 mM L-glutamine (A549, H358 and H292) and incubated at 37°C in a humidified atmosphere with 5% CO_2_. A431 cells were cultured in DMEM high glucose in the presence of 10% FCS. Commercial-grade cetuximab was purchased from Merck. Imgatuzumab (GA201) and the nonglycoengineered version of GA201 (GA201_wt_) were provided by Roche (13). For monoclonal antibody treatments a total concentration of 20 μg/mL was used unless otherwise indicated. Erlotinib (LC Laboratories) and bafilomycin A1 (Sigma-Aldrich) were dissolved in dimethyl sulfoxide (DMSO), stored at −20°C and diluted in fresh medium for use. The final concentration of DMSO never exceeded 0.1% v/v. Recombinant human EGF was obtained from R & D Systems.

### Flow cytometry

Analysis of EGFR expression in the cancer cell lines was performed using flow cytometry. Cells were harvested in phosphate-buffered saline (PBS: 9.7 mM Na_2_HPO_4_, 1.6 mM KH_2_PO_4_, 150 mM NaCl, pH = 7.2) containing 2% FCS (FACS buffer) and left on ice prior to flow cytometry analysis. EGFR membrane expression levels were measured using the anti-EGFR human antibody imgatuzumab or cetuximab at final concentrations of 20 μg/mL in FACS medium. Bound antibody was detected using mouse anti-human FITC (Sigma-Aldrich) diluted 1:50 in FACS medium.

To determine the effects of anti-EGFR monoclonal antibody treatment on EGFR membrane levels, cells were treated with imgatuzumab (20 μg/mL), cetuximab (20 μg/mL) or the combination (10 μg/mL each or 20 μg/mL each) for 24 and 72 hours. Cells were harvested in FACS buffer and left on ice prior to flow cytometry analysis. To determine the internalization of EGFR monoclonal antibodies and membranous turnover of EGFR in tumor cells, cells were stained on ice with the primary antibodies against EGFR (20 μg/mL final concentration). After staining with the primary antibody; 1) cells were washed with ice-cold FACS buffer and incubated with the secondary antibody for 1 hour at 4°C to measure surface expression. 2) Cells were washed with ice-cold FACS buffer, incubated in culture medium at 37°C for 1, 2, or 4 hours and subsequently incubated with the secondary antibody for 1 hour at 4°C to measure non-internalized EGFR-antibody complexes since the secondary antibodies only bind to surface bound EGFR antibody. The lower the amount of non-internalized EGFR-antibody complexes, the more internalization of EGFR-antibody complexes. 3) Cells were washed with ice-cold FACS buffer, incubated in culture medium at 37°C for 1, 2, or 4 hours and subsequently incubated with the primary antibody, followed by the secondary antibody to measure non-internalized, reappeared receptors and possible *de novo* synthesis of receptors. Duplicate samples were measured for each treatment condition, and corrected for background fluorescence and unspecific binding of the secondary antibody. Measurement was performed on a BD FACSCalibur or BD Accuri C6 (BD Biosciences). Data analysis was performed with FlowJo v10 (Tree Star) and surface receptor expression was expressed as mean fluorescent intensity (MFI).

### Western blotting

#### Cell lysis

After appropriate treatment, cells were washed two times with ice-cold PBS and lyzed using ice-cold Mammalian Protein Extraction Reagent (M-PER) (Thermo Scientific) supplemented with phosphatase and protease inhibitors according to supplemented protocol. During the incubation for 60 minutes on ice, cells were scraped and the cellular contents were transferred to microcentrifuge tubes and stored at -20°C until analysis. Protein concentrations were measured using the Bradford protein assay.

### Western blot analysis

Protein samples from total cell lysates (20 or 50 μg) were subjected to electrophoretic separation on a 7.5 or 10% polyacrylamide gel and transblotted onto a polyvinylidine fluoride (PVDF) membrane (Millipore). Blots were blocked at room temperature for 1 hour in Tris-buffered saline (TBS)/Tween 20 (TBS-T) (0.05%) containing 5% bovine serum albumin (BSA) and incubated with: 1:500 rabbit polyclonal anti-EGFR (#2235); 1:500 rabbit monoclonal anti-phospho-EGFR (Tyr1173) (#4407); 1:500 rabbit monoclonal anti-phospho-EGFR (Tyr1068) (#3777); 1:1000 mouse monoclonal antibody anti-phospho-p44/42 MAPK (ERK1/2) (#9106); 1:1000 rabbit polyclonal anti-p44/42 MAPK (ERK1/2) (#9102); 1:1000 rabbit polyclonal anti-phospho-Akt (Thr308) (#9275); 1:1000 rabbit polyclonal anti-Akt (#9272) (Cell Signaling Technology); 1:10000 mouse monoclonal anti-actin (clone C4) (MP Biomedicals). Blots were subsequently washed and incubated with HRP-anti-mouse or HRP-anti-rabbit antibodies at 1:1500 (Dako). Detection was performed using Lumi-Light Western blotting substrate (Roche Diagnostics Nederland B.V.). Images were captured using a digital imaging system (Bio-Rad).

### Immunofluorescence

Imgatuzumab was directly labeled with Dylight 633-NHS ester (Thermo Scientific). The imgatuzumab buffer was exchanged using 30 kDa polyethersulfone 2 mL Vivaspin-2 filters (Sartorius) to 0.9% NaCl, pH was adjusted to 8.5 and a 10-fold excess of DyLight-633 in DMSO was added. After 1 hour of incubation, antibody-dye conjugates were purified from unreacted dye using Vivaspin centrifugal filters.

For the detection of unlabeled anti-EGFR monoclonal antibodies an anti-human Alexa 488 secondary antibody (Life Technologies) was used. The antibodies against endosomal markers, early endosome antigen 1 (EEA1) (#2411, Cell Signaling Technology) and lysosomal-associated membrane protein 1 (LAMP1) (H4A3) (Santa Cruz Biotechnology) at 1:200 were detected using Alexa 647-labeled secondary antibodies (Life Technologies). To investigate endocytic trafficking, H292 and SW-1573 cells were seeded in 24 wells plates on glass cover slides coated with poly-L-lysine (Sigma-Aldrich). After incubation with the anti-EGFR monoclonal antibodies cells were fixed, permeabilized using 0.1% Triton X-100 (Sigma-Aldrich) and blocked with bovine serum albumin. Cells were incubated overnight with primary antibodies against endosomal markers at 4°C and with secondary fluorescent antibodies for 30 minutes at room temperature. For the Lamp1 staining additional cetuximab and/or imgatuzumab were added to visualize total EGFR. After the DAPI staining (Sigma-Aldrich) cells were mounted using Kaiser's Glycerin (Brunschwig Chemie) and examined using a Leica TCS SP8 confocal microscope (Leica, Wetzlar).

### Growth inhibition and migration assays

For the proliferation assays, SW-1573 (2000 cells/well), H292 (2000 cells/well), H322 (8000 cells/well), A549 cells (2000 cells/well) and H358 (2000 cells/well) were seeded in 12 wells plates. Cells were allowed to adhere overnight. Subsequently, cells were treated with the indicated anti-EGFR monoclonal antibodies (20 μg/mL total) in complete medium containing 10% FCS with or without the addition of 1 or 10 ng/mL EGF and incubated for the indicated time period at 37°C.

For the wound healing assays, H292 and A549 cells (both 150,000 cells/well) were seeded in 24 wells plates. After 24 hours cells were serum starved overnight. Next, a scratch was made in the monolayer with a pipette tip, wells were washed with PBS, and cells incubated in serum free medium containing 10 ng/mL EGF with or without the anti-EGFR monoclonal antibodies at 37°C. Scratch area was measured when the scratch was made and after 16 hours for H292 or 24 hours for A549 using Image J software analysis (1.47v).

### ADCC assays

ADCC was measured using the Lactose Dehydrogenase Cytotoxicity Detection Kit (Roche Diagnostics) according to standard protocol as described previously [13]. Briefly, human PBMCs were prepared from healthy blood donors by Ficoll (GE Healthcare) gradient centrifugation. PBMCs were washed in PBS and resuspended in Aim V medium (Life Technologies). Serial dilutions of either imgatuzumab or cetuximab were added to each well of a 96-well plate. The final concentration of the monoclonal antibody combination was the same as in the single monoclonal antibody experiments. ADCC was conducted using an effector:target (E:T) cell ratio of 25:1 and 4 hours incubation at 37°C. All assays were conducted in triplicate.

### CDC assays

CDC was measured using the Lactose Dehydrogenase Cytotoxicity Detection Kit (Roche Diagnostics). Anti-EGFR monoclonal antibodies (10 μg/mL total) were added to 25.000 tumors cells in a 96-well plate. CDC was conducted using a final concentration of 5% freshly drawn human serum and 4 hours incubation at 37°C. Antibody-dependent cytotoxicity without serum was not observed. All assays were conducted in triplicate.

### Statistics

*In vitro* data were assessed for differences with unpaired two-tailed Student's t-test or two-way ANOVA followed by Bonferroni post-test. Results are represented as means ± SD. A *P*-value < 0.05 was considered significant. Statistical analyses were generated using GraphPad Prism software (version 5.0 GraphPad software).

## SUPPLEMENTARY FIGURES


