# Supplementary material for: ADCC responses and blocking of EGFR-mediated signaling and cell growth by combining the anti-EGFR antibodies imgatuzumab and cetuximab in NSCLC cells
Source: Oncotarget. 2017 Apr 17;8(28):45432–46. doi: 10.18632/oncotarget.17139 (PMC5542198; doi:10.18632/oncotarget.17139)
Supplement: Supplementary file 1 [file oncotarget-08-45432-s001.pdf]

ADCC responses and blocking of EGFR-mediated signaling and cell growth by combining the anti-EGFR antibodies imgatuzumab and cetuximab in NSCLC cells

SUPPLEMENTARY FIGURES

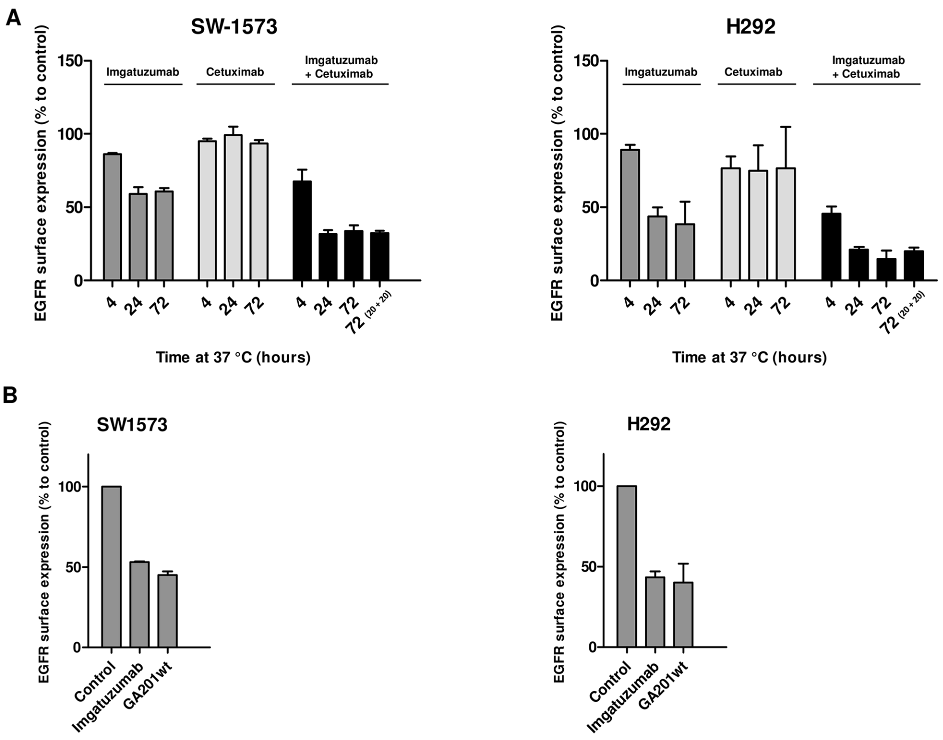

Supplementary Figure 1: Effect of anti-EGFR monoclonal antibody treatment on EGFR surface expression levels. (A) SW-1573 and H292 cells were treated with the anti-EGFR monoclonal antibodies (20 µg/mL total) for 4, 24 and 72 hours, or with 20 µg/mL imgatuzumab combined with 20 µg/mL cetuximab for 72 hours. Surface expression levels were determined using flow cytometry. The surface expression in untreated control cells was set at 100%. All experiments were performed at least twice in duplicate. **(B)** Influence of antibody glycosylation on EGFR surface expression. SW-1573 and H292 were treated with imgatuzumab or $GA201_{wt}$ (20 µg/mL) for 72 hours. Surface expression levels were determined using flow cytometry. The surface expression in untreated control cells was set at 100%. Data points are mean + SD.

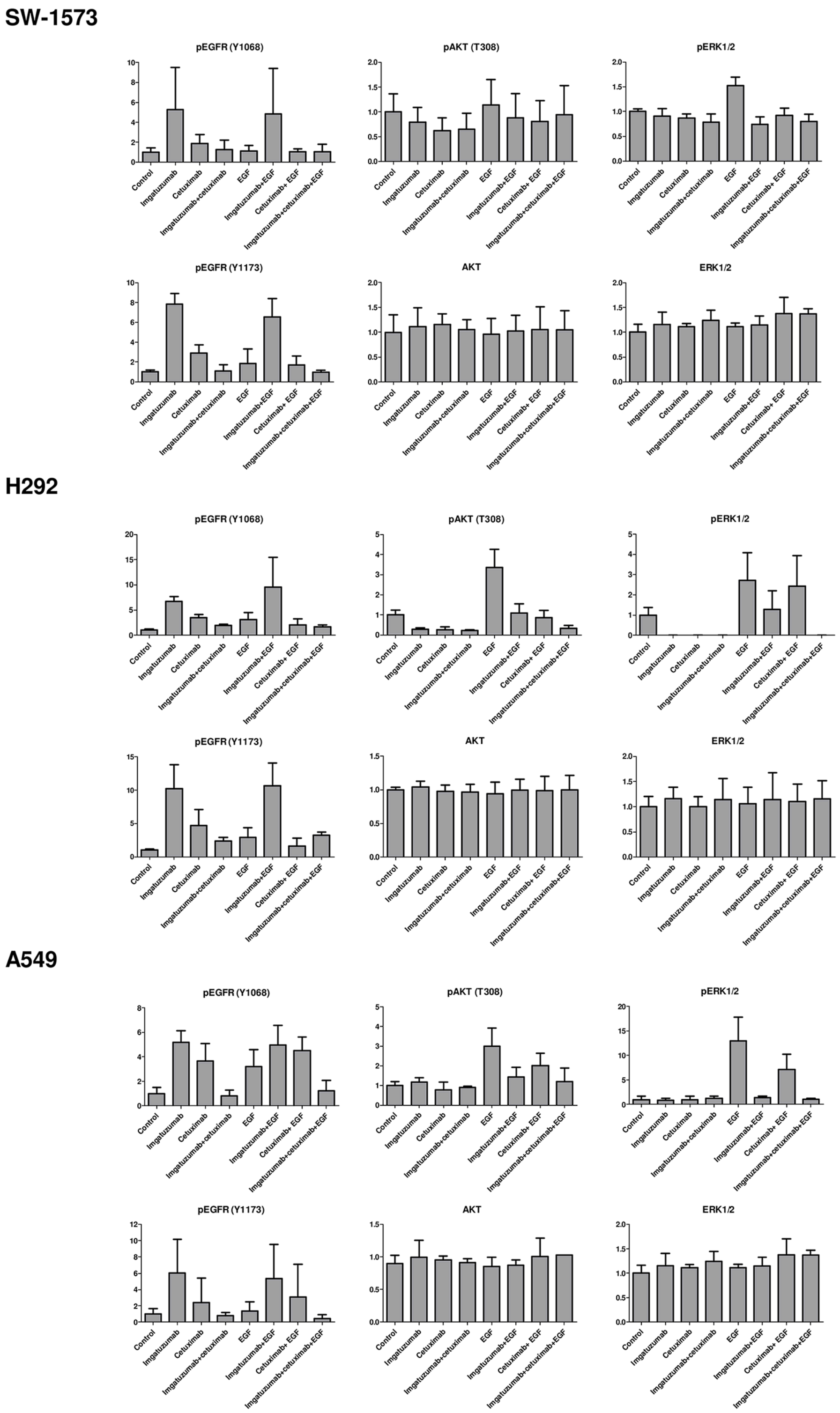

Supplementary Figure 2: Densitometric analysis of the Western blots depicted in Figure 2. The immunoreactive spots were quantified by densitometric analysis and normalized using actin. Values are expressed as fold increase versus control + SD. All experiments were performed in triplicate.

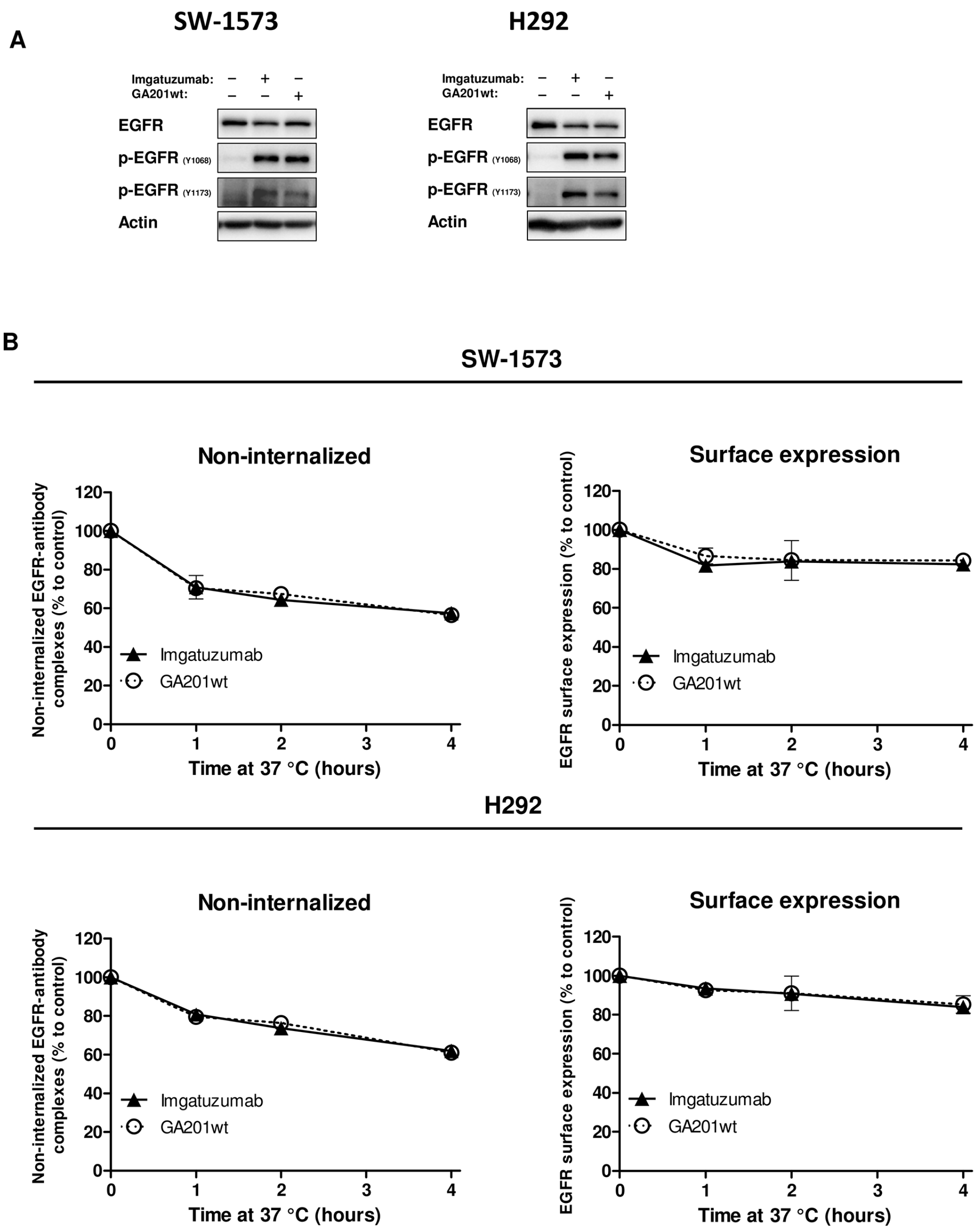

Supplementary Figure 3: Influence of antibody glycosylation on EGFR protein levels and internalization. (A) Western blot analysis of the effect of 24 hours monoclonal antibody treatment on (p)EGFR total protein levels. **(B)** SW-1573 and H292 cells were surface labeled with imgatuzumab or $GA201_{wt}$ on ice and incubated at 37 °C for the indicated times, and then analyzed for the non-internalized EGFR-antibody complexes (left) and cell surface expression (right). The lower the amount of non-internalized EGFR-antibody complexes, the higher the amount of internalized antibody-EGFR complexes. The surface expression at t=0 was set at 100%. The non-internalized and surface expression were determined as described in *Materials and Methods*. All experiments were at least performed twice

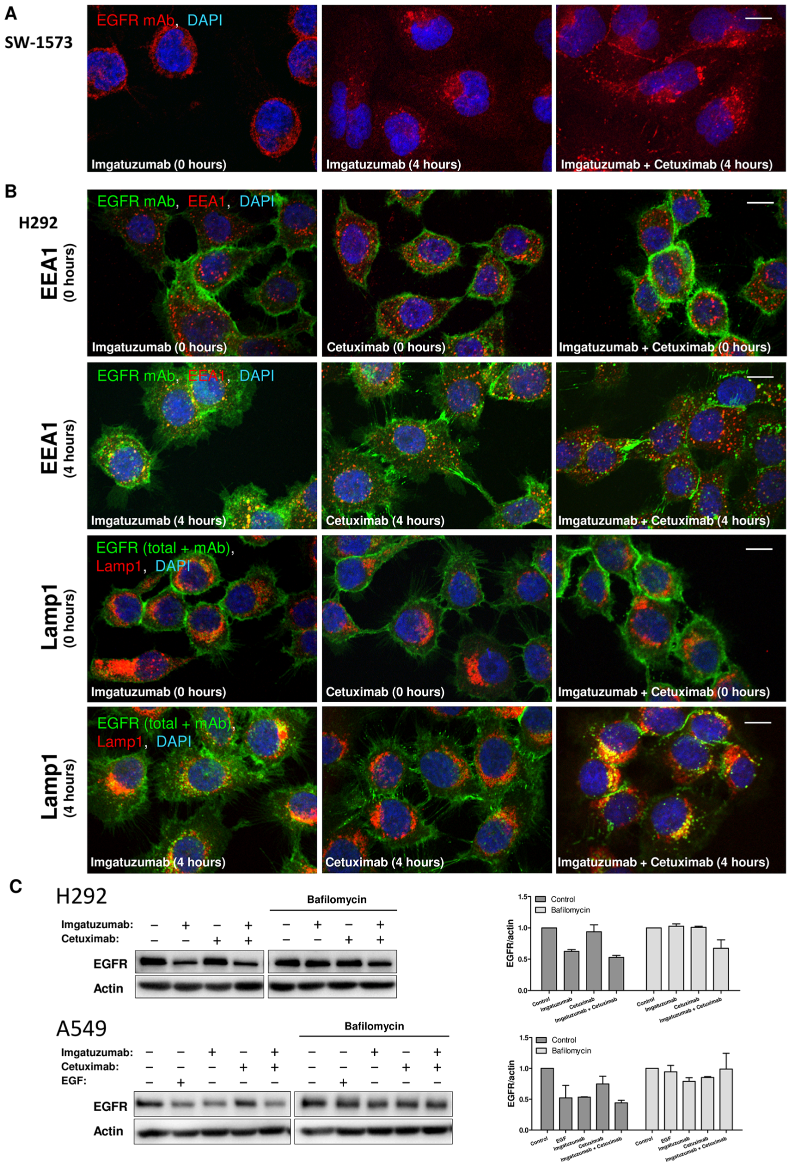

Supplementary Figure 4: Cellular localization of anti-EGFR monoclonal antibodies. (A) fluorescent imaging of the localization of Dylight 633-labeled imgatuzumab with or without cetuximab after 4 hours incubation at 37 ˚C in SW-1573 cells. **(B)** H292 cells were surface labeled with the anti-EGFR monoclonal antibodies on ice and incubated for 4 hours at 37 ˚C. After fixation, permeabilization, and incubation with anti-EEA1 or anti-Lamp1 cells were incubated with fluorescent secondary antibodies (anti-human Alexa 488 against imgatuzumab and cetuximab (green color), Alexa 647-labeled antibodies for EEA1 and Lamp1 (red color). Colocalization appears as yellow (merged images). Images were acquired using a confocal microscope. White scale bars represent 10 µm. **(C)** Western blot analysis of the effect of lysosomal inhibition on monoclonal antibody-induced EGFR degradation. H292 or A549 cells were pretreated for 2 or 16 hours with the lysosomal inhibitor bafilomycin A1 (100 nM). Cells were subsequently treated with the anti-EGFR monoclonal antibodies (20 µg/mL total) or EGF (10 ng/mL) for 4 hours.

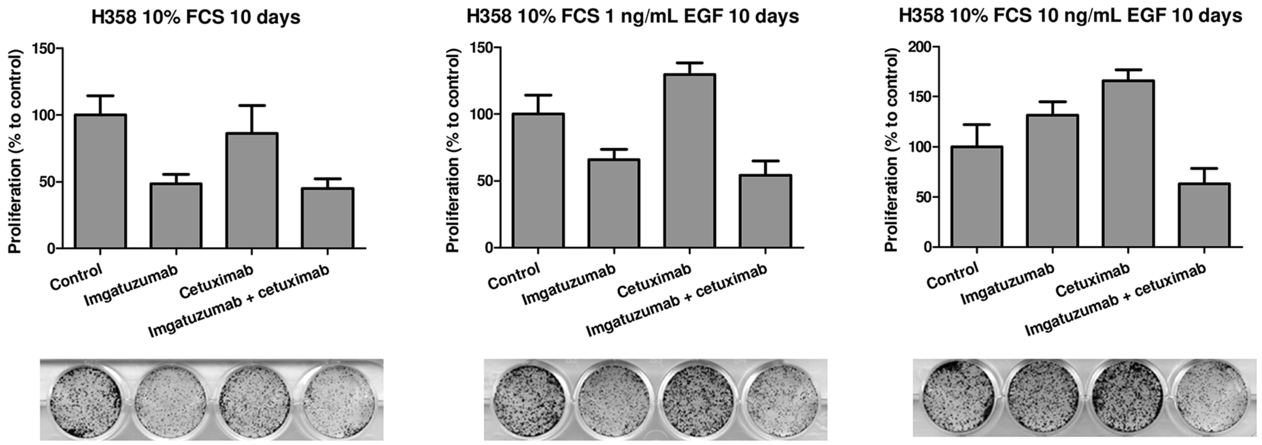

Supplementary Figure 5: *In vitro* growth-inhibitory effect of anti-EGFR monoclonal antibody treatment. Cells were seeded in 12-wells plates and allowed to adhere overnight. Next, H358 cells were treated with the anti-EGFR monoclonal antibodies (20 µg/mL total) for the indicated days under normal growth conditions without additional EGF (medium with 10% FCS) or in medium containing 10% FCS with 1 or 10 ng/mL EGF. Data points are mean + SD. All experiments were performed in triplicate.

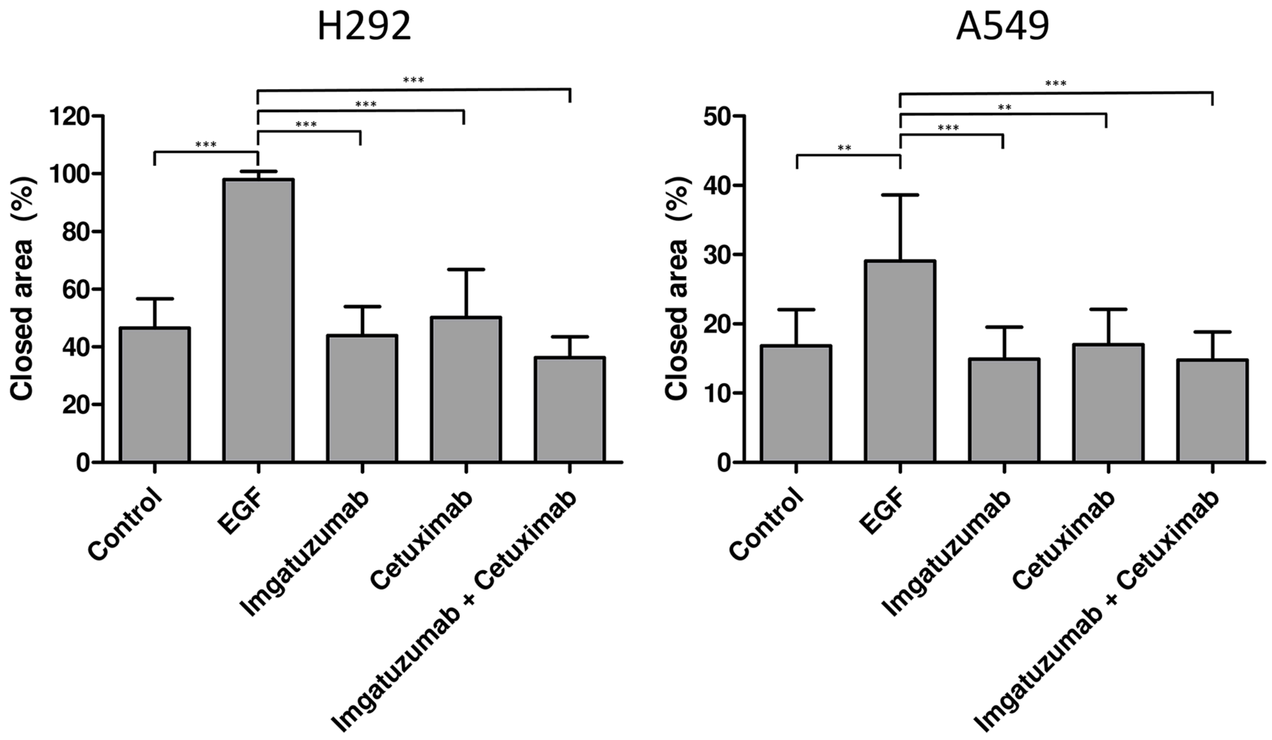

Supplementary Figure 6: Effects of anti-EGFR monoclonal antibody treatment on EGF induced migration. H292 (left) and A549 (right) cells were serum starved overnight. Next, monolayers were wounded with a pipette tip and incubated in serum free medium containing 10 ng/mL EGF with or without the anti-EGFR monoclonal antibodies (20 µg/mL total) at 37 °C. Scratch area was measured when the scratch was made and after 16 hours for H292 and 24 hours for A549. Data points are mean + SD (n = 3). (**$P < 0.01$, ***$P < 0.001$).

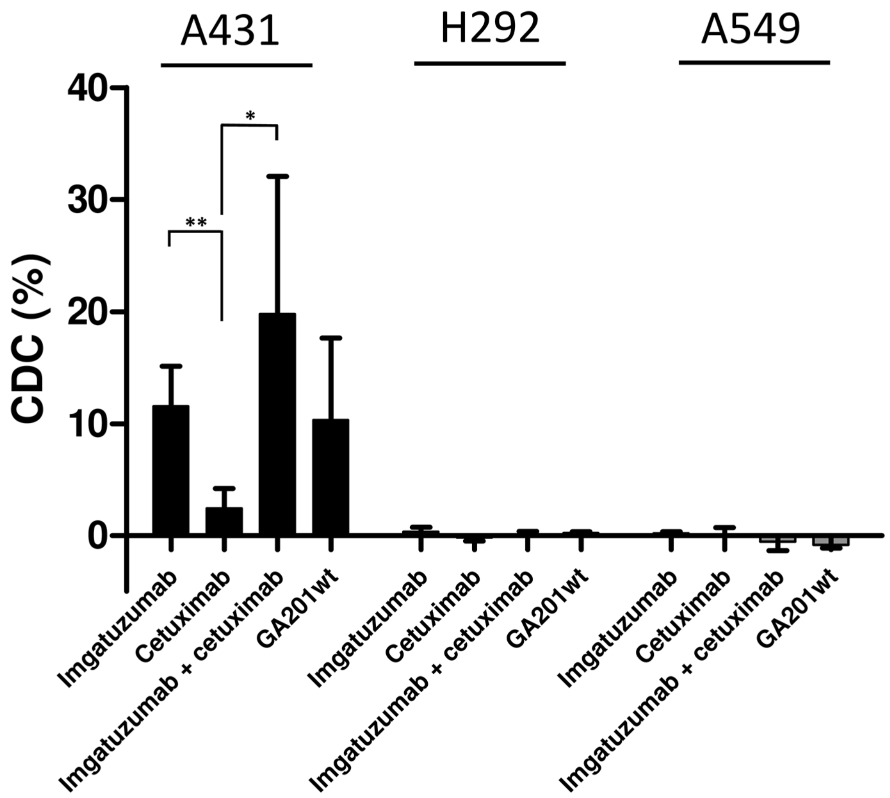

Supplementary Figure 7: CDC analysis of imgatuzumab, cetuximab, the combination or GA201$_{wt}$. Anti-EGFR monoclonal antibodies (10 µg/mL total) were added to A431, H292 and A549 cells. CDC was conducted using a final concentration of 5% freshly drawn human serum and 4 hours incubation at 37 °C. Data points are mean + SD. (**P* < 0.05, ***P* < 0.01). All experiments were performed in triplicate.
